# Comparison of SARS-CoV-2 PCR-Based Detection Using Saliva or Nasopharyngeal Swab Specimens in Asymptomatic Populations

**DOI:** 10.1128/spectrum.00062-21

**Published:** 2021-08-25

**Authors:** Guang Fan, Xuan Qin, Daniel N. Streblow, Cristina Magallanes Hoyos, Donna E. Hansel

**Affiliations:** a Department of Pathology and Laboratory Medicine, School of Medicine, Oregon Health and Science University, Portland, Oregon, USA; b Vaccine and Gene Therapy Institute, Oregon Health and Science University, Beaverton, Oregon, USA; University of Arizona/Banner Health

**Keywords:** SARS-CoV-2, saliva, asymptomatic

## Abstract

The coronavirus disease 2019 (COVID-19) pandemic has challenged clinical diagnostic operations due to supply shortages and high staffing needs to collect nasopharyngeal (NP) swab samples. Saliva is an easily accessible alternative specimen type to overcome some of these challenges. In this study, we first used paired saliva and NP swab specimens (*n* = 128) to compare test performance characteristics with three RNA extraction platforms, i.e., Maxwell RSC (Promega), MagNA Pure 96 (Roche), and KingFisher Flex (Thermo Fisher Scientific), together with two PCR chemistries, i.e., severe acute respiratory syndrome coronavirus 2 (SARS-CoV-2) (2019-nCoV) Centers for Disease Control and Prevention (CDC) quantitative PCR (qPCR) probe assay (Integrated DNA Technologies) and TagPath COVID-19 combination kit (Thermo Fisher Scientific). This study demonstrated that both saliva and NP swab specimens performed well, with 97% agreement when tested by the CDC qPCR chemistry using Maxwell and MagNA Pure RNA extraction platforms. We then compared 12 weeks of saliva and NP swab testing results using two independent asymptomatic populations, including a community surveillance program using saliva samples only (*n* = 466) and preoperative screening using NP swab samples only (*n* = 8,461). The positive detection rates among participants with either saliva or NP swab samples were 1.07% and 1.12%, respectively, which confirms the low pretest probabilities for COVID-19 infections in asymptomatic populations. Notably, there was no increased proportion of low-titer cases (inconclusive results) reported in the asymptomatic groups, compared with the all-comers groups (0.21% and 0.66%, respectively, in the community population and 0.25% and 0.49%, respectively, in the preoperative population); this suggests that low-viral-titer carriers can be found similarly in both groups with saliva or NP swab specimens. In summary, saliva can be considered a good alternative for noninvasive but well-instructed self-collection.

**IMPORTANCE** Our study shows that saliva is a noninvasive respiratory secretion sample type that contains equal or more host materials (RNase P), compared with those contained in the corresponding NP swab specimens, in 103 paired samples. SARS-CoV-2 detection with two RNA extraction platforms, Maxwell and MagNA Pure, with CDC qPCR chemistry showed similar test sensitivities for paired specimens. We then analyzed SARS-CoV-2 detections rates in two independent groups of asymptomatic participants, i.e., a group at a community screening station with supervised saliva collection only (*n* = 466) and a preoperative screening group (*n* = 8,461) with NP swabbing only. Similar detection rates of 1.07% for the community group and 1.12% for the preoperative group supported the similar test performances in these groups predicted to have low pretest probabilities of infection. With mindful preparation, saliva can be considered for schools and clinical participants when adequate collection education can be provided and compliance can be established.

## INTRODUCTION

The coronavirus disease 2019 (COVID-19) pandemic has impacted society and human behavior in many significant ways, from as individualized as mask wearing and social distancing to as broad as the complete lockdown of cities. With the increased testing needs associated with this robust public health response, clinical laboratories have faced unprecedented high-volume testing requests while encountering supply shortages and rationing of reagents (1). Many laboratories have resorted to bringing on multiple RNA extraction platforms and PCR chemistries for severe acute respiratory syndrome coronavirus 2 (SARS-CoV-2) testing in order to meet the ever-increasing demand and to provide flexibility in the setting of needing specific reagents while encountering supply shortages.

The nasopharyngeal (NP) swab sample has been widely used as the diagnostic specimen of choice. However, its dependence on qualified health care professionals for collection and ongoing shortages of NP swabs and viral transport medium have limited testing capacity during the current pandemic.

A number of studies have analyzed paired saliva and NP swab PCR testing results for patient testing or campus screening. Those studies reported an overall trend of lower PCR cycle threshold (*C_T_*) values using NP swab samples, compared to saliva samples (2, 3), while a few studies reported no significant differences (4–6). The use of saliva samples has remained a variable practice for either diagnostic or surveillance operations to date, without clear professional guidelines.

Asymptomatic carriers have accounted for viral transmission since the early days of the COVID-19 pandemic. One study reported 3% asymptomatic cases in health care workers among 1,000 staff participants at a large UK hospital in April 2020 (7). In a meta-analysis and literature review using 94 PubMed peer-reviewed studies between March and June 2020, the authors drew an overall estimate of 20% (range, 3 to 67%) asymptomatic cases among SARS-CoV-2-infected people (8). All of those studies focused invariably on the PCR-positive cohort, which is often enriched in symptomatic individuals, to estimate the subset of asymptomatic carriers. To date, there is a lack of information regarding studies that use a broader population for COVID-19 prevalence, especially among those who otherwise have no known exposure or no clinical signs and symptoms for COVID-19 infections at the time of testing.

Our study first sought to validate PCR-based testing results for saliva samples, in comparison to NP swab results, using three RNA extraction platforms, including Maxwell RSC (Promega Corp.), MagNA Pure 96 (Roche Diagnostics), and KingFisher Flex (Thermo Fisher Scientific), and two PCR chemistries, including the Centers for Disease Control and Prevention (CDC) SARS-CoV-2 quantitative PCR (qPCR) probe assay (Integrated DNA Technologies, Inc.) and the TagPath COVID-19 multiplex qPCR probe set (Thermo Fisher Scientific). The second aim was to confirm the utility of saliva samples for diagnostic and surveillance use by retrospectively comparing PCR testing data for a large number of saliva and NP swab specimens from asymptomatic populations using the same mixed testing platforms.

## RESULTS

### Comparison of SARS-CoV-2 PCR results from directly matched saliva and NP swab specimens.

We compared 128 paired saliva and NP swab specimens from patients 4 to 67 years of age (female patients, 73/128 patients [57%]) for SARS-CoV-2 PCR test results. All sample pairs were maximally analyzed, as quantities allowed, by combinations of all three RNA extraction platforms and two PCR chemistries (Table 1). Nineteen paired saliva and NP swab samples tested positive and concordant, while 101 paired samples were all negative. Two pairs of samples showed a single inconclusive detection of three platform-chemistry combinations for the saliva sample only, and two pairs showed a single inconclusive detection of three platform-chemistry combinations for the NP swab sample only. These four pairs were excluded from analysis, since either the saliva specimen or the NP swab specimen in the pair showed inconclusive PCR results by one of the testing platforms and was negative by all other testing methods. Therefore, the percent test agreement for combined detected and not detected findings was 97% between saliva and NP swab specimens by the mixed use of three extraction methods and two PCR chemistries in this comparison.

**TABLE 1:** *C_T_* values according to RNA extraction platform and PCR targets for direct paired positive saliva and NP swab samples used for test validation

RNA extraction and PCR methods and sample type	*C_T_* (median [range]) for target:					*P* (*t* test)
	N1	N2	S gene	N gene	ORF1ab	
Maxwell RNA extraction and CDC PCR assay (*n* = 13 pairs)						0.42^*a*^
Saliva	23.88 (16–33)	24.22 (11–38)				
NP swab	21.18 (11–36)	22.46 (17–34)				
MagNA Pure RNA extraction and CDC PCR assay (*n* = 15 pairs)						0.22^*a*^
Saliva	25.63 (19–34)	26.26 (18–35)				
NP swab	22.84 (12–25)	22.28 (12–40)				
KingFisher Flex RNA extraction and CDC PCR assay (*n* = 6 pairs)						0.03^*a*^
Saliva	25.84 (17–25)	26.67 (17–29)				
NP swab	17.15 (13–27)	17.33 (13–27)				
KingFisher RNA Flex extraction and multiplex assay (*n* = 15 pairs)						0.006^*b*^
Saliva			28.00 (18–35)	27.44 (18–34)	26.28 (10–29)	
NP swab			24.56 (12–30)	24.80 (12–29)	23.97 (17–32)	

a N1 target.

b N gene target.

**FIG 1:**
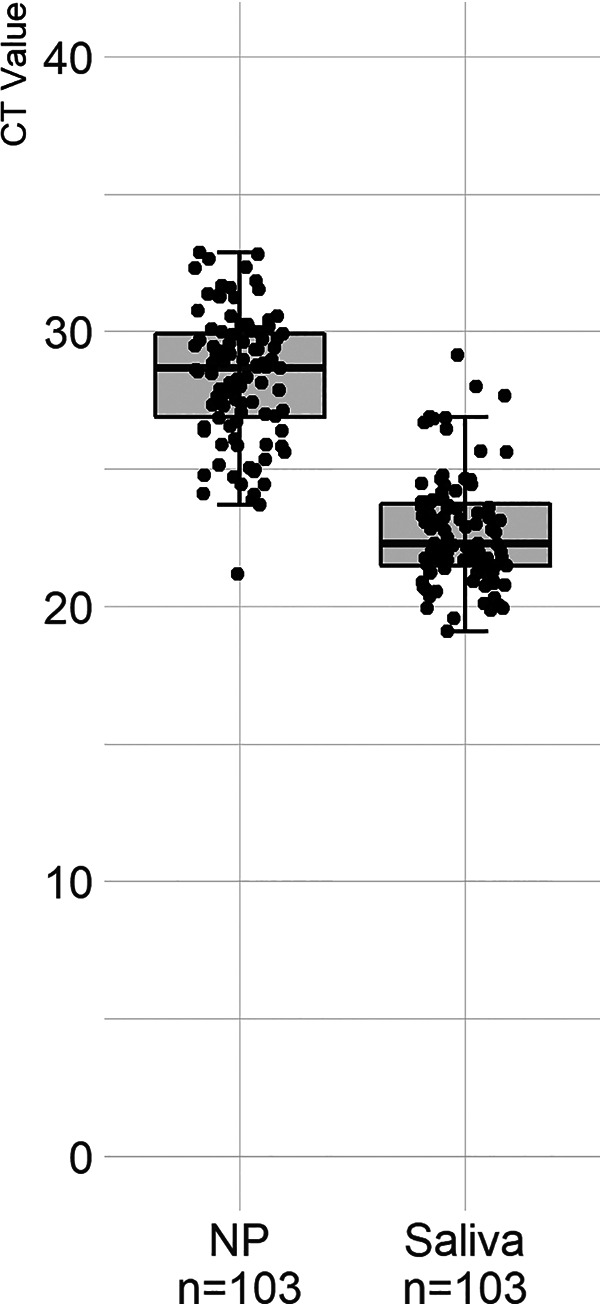
Comparison of *C_T_* value distributions for RP detection in paired saliva and NP swab specimens.

**FIG 2:**
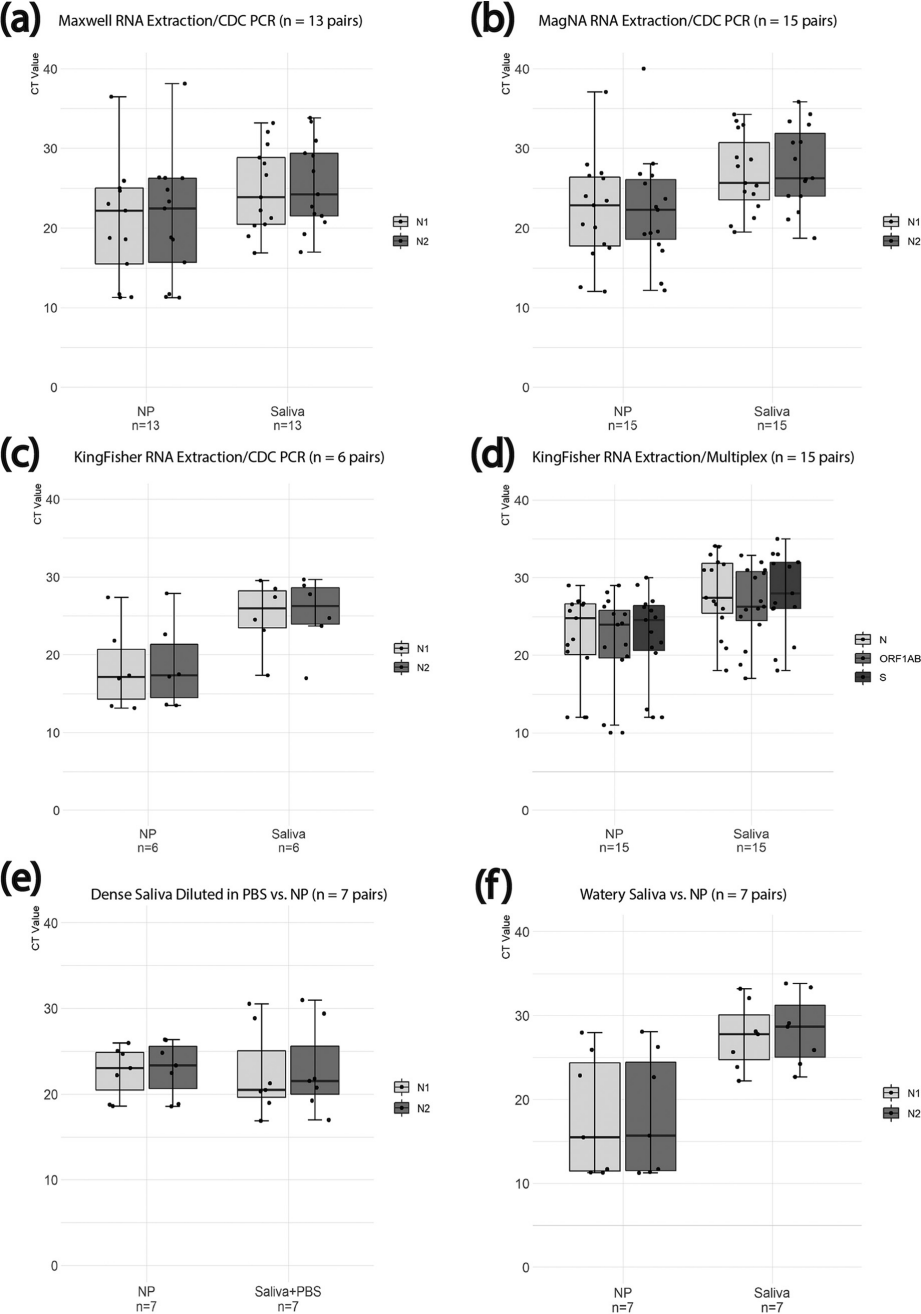
Comparison of PCR *C_T_* values between paired saliva and NP swab specimens that were tested with combinations of three RNA extraction platforms and two PCR chemistries. (a) Maxwell RNA extraction and CDC PCR assay (*n* = 13 pairs). (b) MagNA Pure RNA extraction and CDC PCR assay (*n* = 15 pairs). (c) KingFisher Flex RNA extraction and CDC PCR assay (*n* = 6 pairs). (d) KingFisher RNA extraction and KingFisher TaqPath multiplex PCR assay (*n* = 15 pairs). (e) Dense saliva specimens diluted with PBS versus NP swab specimens (*n* = 7 pairs). (f) Watery saliva samples versus NP swab specimens (*n* = 7 pairs).

We further evaluated the quality of these two specimen types by measuring human RNase P (RP) detection. The median *C_T_* value for RP from saliva samples appeared to be lower than that from NP swab samples in 103 paired specimens tested for RP as the internal control (*P* = 5.26 × 10^−36^) (Fig. 1). The findings suggested that there were increased levels of host RNA detected in saliva samples, compared to NP swab samples. In contrast, the median *C_T_* value based on the viral N target from saliva specimens appeared to be higher than that from NP swab specimens in the 19 paired positive samples, independent of the three extraction methods and two PCR chemistries (Table 1 and Fig. 2a to d). The trend appeared to suggest that the presence of viral templates in saliva specimens was lower than that in NP swab specimens; however, there was no statistically significant difference, based on *C_T_* values generated, between the two sample types using the CDC PCR assay with either Maxwell (*P* = 0.42) or MagNA Pure (*P* = 0.22) RNA extraction methods (Table 1). When KingFisher Flex RNA extraction was used, however, statistical differences were observed between *C_T_* values for the two specimen types using either CDC PCR (*P* = 0.03) or Fisher multiplex PCR (*P* = 0.006) (Table 1). With these findings, subsequent saliva samples were tested only by using Maxwell or MagNA Pure RNA extraction, followed by the CDC PCR assay.

RNA extraction was difficult for 7 dense saliva samples. For those 7 samples, we added 500 μl of phosphate-buffered saline (PBS) to each estimated ∼1 ml of saliva (labeled saliva + PBS in Fig. 2e) and vortex-mixed the samples well. These 7 saliva plus PBS samples and a comparator 7 watery saliva samples without PBS were tested against their corresponding NP swab samples. Paired results with MagNA Pure with CDC PCR chemistry are shown as an example in Fig. 2e and f. The saliva plus PBS samples produced similar *C_T_* results, compared to those of their paired NP swab samples (*P* = 0.95) (Fig. 2e). In contrast, the 7 watery saliva samples without PBS produced rather distinct *C_T_* results, compared to those of their NP swab samples (*P* = 0.014) (Fig. 2f). This finding suggests that the addition of PBS to dense saliva specimens can improve overall nucleic acid extraction without compromising viral RNA recovery and detection. As speculated, watery saliva samples may be indicative of nonoptimal sample collection, which might have contributed to overall higher viral detection *C_T_* values in pairwise comparisons with NP swab samples (Fig. 2b to d and f).

### Retrospective analysis of saliva and NP swab specimen test performances in two asymptomatic populations.

**FIG 3:**
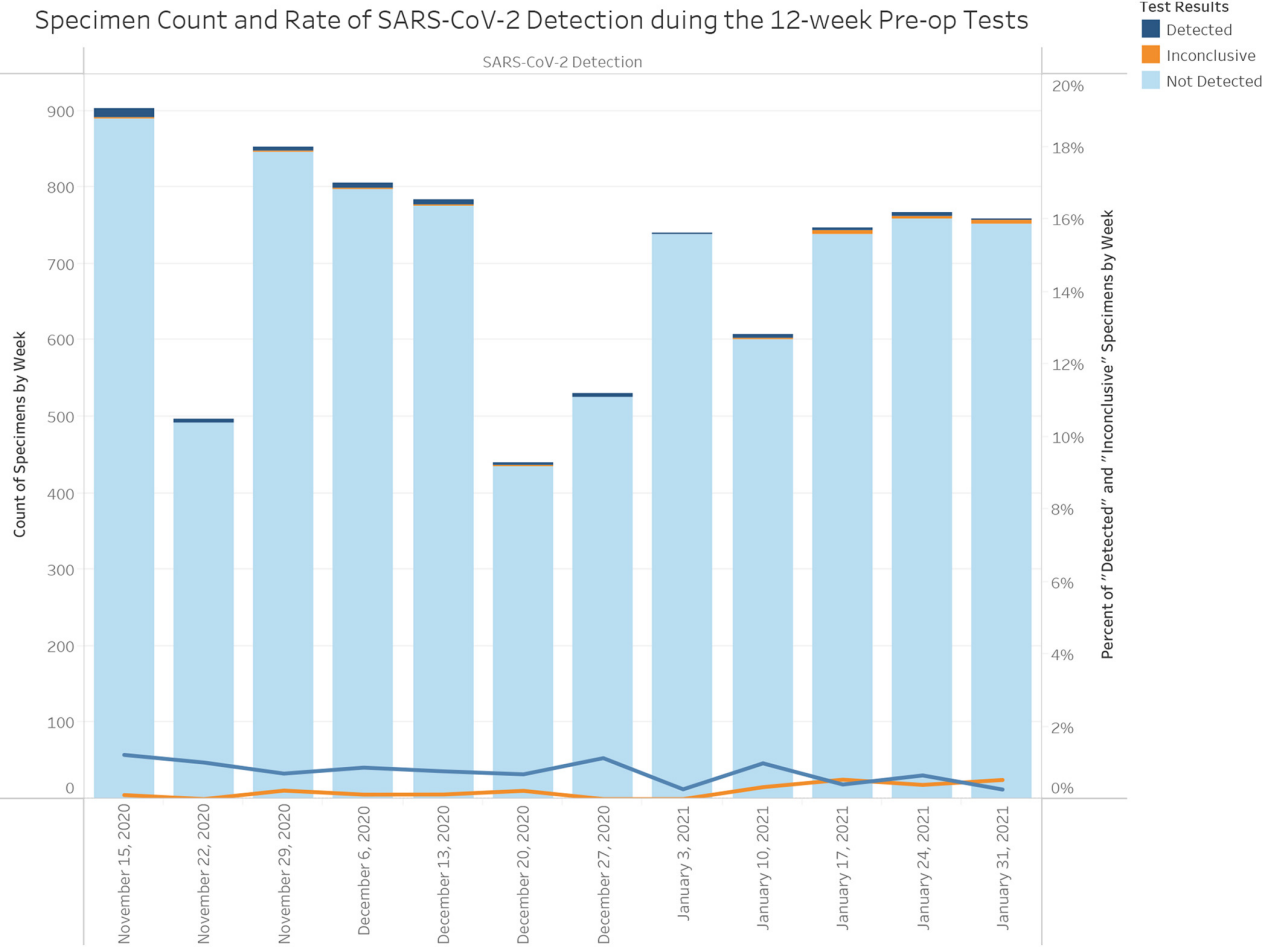
Preoperative screening results for asymptomatic patients using NP swab samples over the 12-week period.

**FIG 4:**
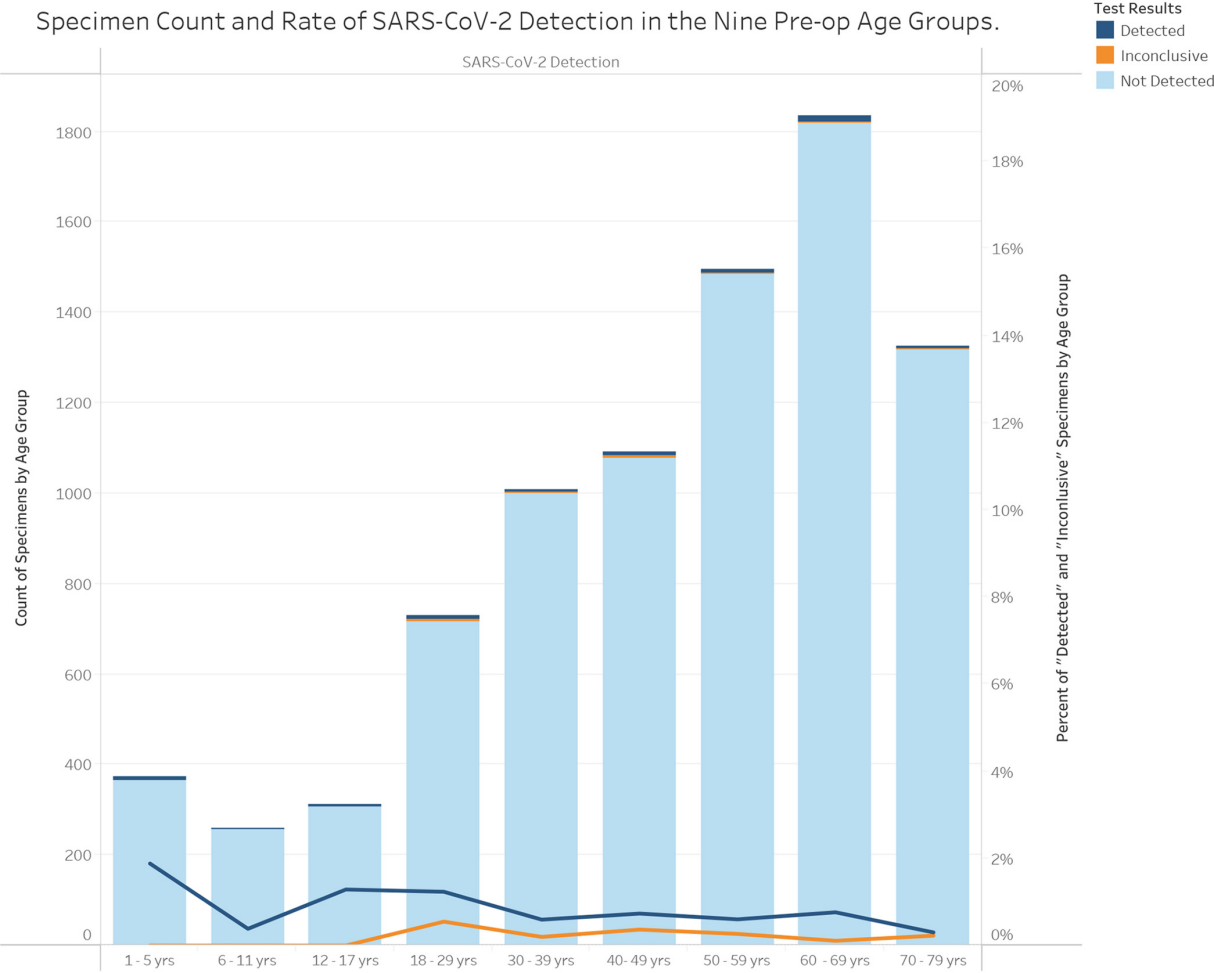
Preoperative screening results for asymptomatic patients using NP swab samples, separated into nine age groups, over the 12-week period.

We analyzed the testing results for two independent asymptomatic populations, including one cohort from a community surveillance station, which was tested using saliva samples only (*n* = 466; female, *n* = 288 [61.8%]), and a second cohort from our health care system preoperative screening stations, which was screened using NP swab samples only (*n* = 8,461; female, *n* = 4373 [51.9%]). The community asymptomatic cohort samples were processed by Maxwell or MagNA Pure RNA extraction with CDC PCR only, while the preoperative screening samples were completed with mixed use of all three RNA extraction methods and two PCR chemistries. Using matched age ranges, a total of 12 weeks of data aggregates were included in this comparison (Fig. 3 and 4).

Among the 466 saliva specimens collected at the community screening site from asymptomatic individuals, there were 5 positive findings, including 1 inconclusive result, resulting in a 1.07% positive rate in the asymptomatic population (Table 2). In comparison, the 8,461 NP swab specimens collected from asymptomatic individuals in preoperative screening showed 95 positive findings, including 21 inconclusive findings, which resulted in a 1.12% positive rate among asymptomatic individuals (Table 2). Figure 3 illustrates the PCR findings for the preoperative test group over the 12-week period. Figure 4 shows the same PCR findings distributed by age groups, in which a relatively higher detection rate of 1.88% in the age group of 1 to 5 years was notable. Similar patterns were observed in the asymptomatic community cohort (data not shown due to small sample size).

**TABLE 2:** SARS-CoV-2 detected and inconclusive findings using community saliva testing, compared to hospital NP swab testing

Test result	Community asymptomatic group (saliva samples)	Community all-comers group (saliva samples)	Hospital asymptomatic preoperative group (NP swab samples)	Hospital all-comers group (NP swab samples)
No. detected	4	55	74	7,892
No. inconclusive	1	4	21	373
No. of positive samples	5	59	95	8,265
Total no. of samples	466	607	8,461	76,297
Proportion of positive findings (%)	1.07	9.72	1.12	10.83
Proportion of inconclusive results in total samples (%)	0.21	0.66	0.25	0.49

### Comparison of saliva and NP swab results between asymptomatic and all-comers SARS-CoV-2 testing populations.

Finally, we compared the ability of the saliva test to mirror percent positive results seen with NP swab testing, which also included their corresponding overall positive rates associated with the community all-comers (*n* = 607) versus the hospital all-comers (*n* = 8,461) matched by age and week (Table 2). The test period of this study encompassed the winter COVID-19 case surge, when the average positive rates for the all-comers groups were similar at 9.72% and 10.83% for the community all-comers and the hospital all-comers, respectively (Table 2). The weekly positive rates for preoperative hospital patients were similar at ∼0.26 to 1.22% during the 12 weeks (Fig. 3). The weekly positive rates for the hospital all-comers cohort ranged from as high as 13.08% in the fourth week of November 2020 to as low as 4.25% in the first week of February 2021 (data not shown). Moreover, inconclusive results that suggested low viral titers near the analytical limit of detection and the proportions of inconclusive results were similar in the asymptomatic groups and the corresponding all-comers comparators (0.21% and 0.66%, respectively, for the community group and 0.25% and 0.49%, respectively, for the hospital group) (Table 2).

## DISCUSSION

In this study, we compared test performance characteristics between saliva and NP swab specimen types, using a variety of molecular extraction platforms and PCR chemistries. Our validation study showed that the two sample types had similar analytical performance characteristics, with 97% result agreement over 124 paired specimens. When *C_T_* values for the SARS-Co-V-2 PCR results were compared, saliva samples produced a trend of lower viral levels (higher *C_T_* values), compared with the paired NP swab samples, but the difference was not statistically significant using the CDC nonmultiplex PCR chemistry with Maxwell and MagNA Pure extraction. A statistical *C_T_* difference between the two sample types was associated only with KingFisher extraction and the multiplex PCR method; therefore, KingFisher RNA extraction and multiplex PCR were not used for saliva sample testing. It is possible that the reduced PCR sensitivity with the KingFisher platform largely suffered from multiplex PCR chemistry (9), while reduced RNA extraction efficiency with CDC PCR chemistry could have been due to too few samples (*n* = 6) included in the comparison (Table 1).

Similarly, we determined that addition of PBS to dense saliva specimens yielded usable specimens that showed *C_T_* values similar to those of their paired NP swabs. One possibility is that the dense saliva samples may represent deeper airway materials, resulting in higher viral yields (10). In contrast, the 7 watery saliva samples showed significantly higher *C_T_* values for viral detection, compared to their NP swab samples (5). In fact, the 7 watery saliva samples represented more than one-third of the positive viral saliva samples (7/19 samples) and thus contributed to their overall higher *C_T_* values (Table 1 and Fig. 2a to f). With these findings, our study confirms that specimen quality rather than specimen type is more important for the analytical sensitivity of the test. It further strengthens the notion that saliva can be a reliable specimen alternative for SARS-CoV-2 testing when well-instructed self-collection can be performed and when optimal RNA extraction and PCR chemistry have been determined.

As problematic as the term asymptomatic can be, viral infections prior to symptom onset (presymptomatic) or with uncharacteristic and subclinical symptoms (paucisymptomatic) cannot be definitively ruled out (11). Many studies have examined SARS-CoV-2 PCR-positive cases for those that do not meet the commonly used clinical criteria for COVID-19 infections and have used those findings to predict the role of carriers in the spread of viruses in the community. While those studies provide valuable information on individuals who received testing in accordance with high pretest probability through potential exposures, such as cruise ship and health care workers (8, 12), there are currently no analyses of viral prevalence in people who otherwise had low likelihood of exposure and were seeking health care attention for other medical needs.

This study attempted to address the proportion of asymptomatic viral carriers in the local population by using SARS-CoV-2 PCR test data generated from a high-throughput laboratory during a period with high COVID-19 prevalence, from 15 November 2020 to 6 February 2021. Specifically, the analysis included two independent data sets from groups that were matched by age range and testing time frame. The participants in the two asymptomatic test categories were predicted to have low pretest probability of COVID-19 infection and thus functioned as a subset of their corresponding all-comers cohort. Saliva and NP swab samples produced similar positive findings of about 1 in 100 individuals (1.07% and 1.12%, respectively). Moreover, the weekly rates of positive findings were very similar during the 12 weeks, as were the positive rates among the age groups. The similar viral hit rates among all age groups suggested that viral transmission paths leave no age exception (Fig. 4), including the very young (1 to 5 years of age). The finding confirms a notable estimate based on a machine learning model with a prevalence prediction of 0.6% in early and mid-January 2021 in Oregon (13, 14).

In contrast, for the patient populations containing those who were considered all-comers in the community group and the hospital group (*n* = 607 and *n* = 76,297, respectively), positive findings were on average 9.72% and 10.83%, respectively, during the analysis period. Notably, there were no increased proportions of inconclusive cases in the asymptomatic groups, compared with the all-comers groups (0.21% and 0.66%, respectively, in the community population and 0.25% and 0.49%, respectively, in the preoperative population), which suggests that carriers with low viral titers can be found similarly in both groups using either saliva or NP swab sample testing.

The retrospective positive viral rate of 1.12% in the preoperative screening test group was among a sizable patient population (*n* = 8,461), using established preoperative test conditions with minimal selection bias. The community outreach testing program was set forth with very broad inclusion criteria concerning prior travel, family visits, unknown exposure, and just informational testing. We think that the preoperative patients are highly representative of asymptomatic individuals who otherwise would not seek medical attention for their COVID-19 infections. Therefore, we think this is a first report of SARS-CoV-2 PCR findings for a sizable asymptomatic cohort of individuals who could serve as a proxy for population-level estimates of COVID-19 prevalence during the 12-week period of 15 November 2020 to 6 February 2021 in a large metropolitan area. However, there are major limitations to this analysis, as the sample types are completely different for the two asymptomatic cohorts. Without direct paired samples from both cohorts, these data comparisons remain retrospective observational field checks. Future in-depth studies in which diverse saliva qualities can be controlled are necessary.

In conclusion, this study shows that saliva is a convenient alternative specimen type, compared to NP swab specimens, based on test validation and use in community screening, compared to preoperative screening tests. Although the sample size in the saliva group for the community surveillance is relatively small, the participants were screened by health care professionals for the absence of clinical signs and symptoms suggestive of COVID-19 infections and the program was promoted as no-barrier community testing. The similar rates of SARS-CoV-2 detection of 1.07% in saliva samples and 1.12% in NP swab samples confirmed that selection bias did not appear to impact results in asymptomatic populations. This retrospective analysis comparing two sample types from two unique testing cohorts suggested that the mixed PCR test modalities yielded similar analytical characteristics. With these promising findings, we would still like to offer a cautionary reminder: the use of saliva samples for PCR testing should not be generalized or be widely adopted to replace NP swab samples. As shown in our data, even with supervised collection, the variability of *C_T_* values between watery and dense saliva samples was large. The use of specialized saliva collection devises also played a role in quality specimen collections. With mindful preparation, saliva samples can be considered for schools and clinical participants when adequate collection education can be provided and compliance can be established.

## MATERIALS AND METHODS

### Saliva and NP swab sample collection and storage condition.

This project was approved by the institutional review board (IRB) at Oregon Health and Science University (OHSU). We collected 128 paired saliva and NP swab specimens from consenting individuals. For the collection of saliva samples, individuals were instructed by health care professionals using the SalivaBio saliva collection aid (Salimatrics, USA) to gently express salivary secretions into a sterile tube, emphasizing >30 min after food or fluid intake. A minimum of 1 ml was the required volume. Saliva specimens were kept at the collection site at an ambient temperature of 15°C to 25°C for <3 h and were then transported to the OHSU COVID-19 diagnostic laboratory and stored in a refrigerator at 2°C to 8°C for <24 h before PCR analysis. To ensure adequate RNA extraction from dense saliva samples, 500 μl of PBS was added and mixed well before RNA extraction. NP swab samples were collected by trained medical personnel following the standard collection procedure.

### RNA extraction and PCR analysis.

RNA extraction was performed with three different extraction methods, including Maxwell RSC, MagNA Pure 96, and KingFisher Flex, following the manufacturers’ instructions. For RNA extraction, a sample volume of 300 μl was used for the Maxwell platform and a sample volume of 200 μl was used for the MagNA Pure and KingFisher Flex platforms. A 50-μl RNA elution volume was obtained from each extraction method. RNA samples were tested by two PCR chemistries, i.e., the 2019-nCoV CDC emergency use authorization (EUA) kit containing a N1, N2, and human RP primer/probe mix (Integrated DNA Technologies, Inc.) and the TaqPath multiplex reverse transcription (RT)-PCR COVID-19 kit containing N gene, S gene, and open reading frame 1ab (ORF1ab) primers and MS2 phage control (Thermo Fisher Scientific). TaqPath one-step RT-PCR master mix (Applied Biosystems) was used for the CDC kit, and TaqPath one-step multiplex master mix was used for the TagPath kit. A 5-μl RNA extract was used for both PCR chemistries. All PCR tests were performed using a QuantStudio5 thermocycler (Thermo Fisher Scientific) with a limit of detection of ∼15 copies/reaction for all mixed uses of extraction and PCR platforms.

Each sample pair was tested in parallel on multiple platforms with the same lot and preparations of positive and negative controls being used for each run. The same control materials were used across all testing platforms throughout the RNA extraction and PCR processes.

### PCR result interpretation and report.

A PCR test was considered valid if the internal control RNA (either RP or MS2) was detected and reported. An invalid result was reported when the PCR was nonreactive to the internal control target and nonreactive to any of the viral targets. Similar interpretive criteria were adopted for the SARS-CoV-2 PCR tests that were determined by using more than one viral target in the SARS-CoV-2 (2019-nCoV) CDC qPCR probe assay (Integrated DNA Technologies) and the TagPath COVID-19 combination kit (Thermo Fisher Scientific). A detected result was reported when two or three viral targets were reactive, whereas an inconclusive result was reported when a single viral target was reactive. In analyses of retrospective data for asymptomatic and all-comers patients, both detected and inconclusive results were treated as SARS-CoV-2 positive. A not detected result was reported when the PCR was not reactive to any of the viral targets. When the effectiveness of the mixed use of analyzers was assessed, PCR *C_T_* values from the viral N1 target in the CDC chemistry and the N gene target in the TaqPath chemistry were used for measurement, based on their consistent performance for the 19 positive sample pairs (15).

### Data analysis.

First, the test performance characteristics for the 128 paired specimen types (saliva and NP swab specimens) were analyzed for concordant and discordant test results using the aforementioned two PCR chemistries and three RNA extraction platforms. Statistical analyses comparing mean *C_T_* values for either PCR chemistry downstream from the three extraction platforms were performed using Microsoft Excel *t* test analysis.

A retrospective SARS-CoV-2 PCR clinical testing data analysis was carried out following the laboratory analytical test characterization, comparing the paired specimen types. SARS-CoV-2 PCR clinical testing results were then compared between two unrelated asymptomatic cohorts whose specimens were either saliva or NP swab samples. All PCR data used for this analysis were matched by participants’ age range (1 to 80 years) and testing period (15 November 2020 to 6 February 2021). The first cohort included 466 saliva specimens collected from asymptomatic individuals participating in a community screening program that welcomed both symptomatic and asymptomatic individuals, with sample collection performed by qualified health care professionals using the CDC criteria (16). The second cohort included 8,461 NP swab specimens collected from patients who were tested as part of our preoperative COVID-19 screening program. Both study groups were considered asymptomatic based on their low pretest probability for COVID-19 infections at the time of the test and the absence of self-reported symptoms on a screening questionnaire. The corresponding data for all-comers reference groups included community all-comers (*n* = 607, including the asymptomatic community cohort) and OHSU Hospital all-comers (*n* = 76,297, including the preoperative cohort), regardless of the subjects being symptomatic or not. While the community all-comers group included the use of both saliva and NP swab specimens, the OHSU Hospital all-comers group included primarily NP swab samples (>99%), with a relatively small number of laboratory-validated bronchoalveolar lavage or tracheal aspirate samples and nasal or oral swab specimens. The PCR inconclusive samples were included as part of the positive findings based on repeat testing, according to our institution’s standard recommendations. Tableau was used as a tool for data analysis and visualization.
